# Abundance of Major Cell Wall Components in Natural Variants and Pedigrees of *Populus trichocarpa*

**DOI:** 10.3389/fpls.2022.757810

**Published:** 2022-02-03

**Authors:** Anne E. Harman-Ware, Renee M. Happs, David Macaya-Sanz, Crissa Doeppke, Wellington Muchero, Stephen P. DiFazio

**Affiliations:** ^1^Renewable Resources and Enabling Sciences Center, Center for Bioenergy Innovation, National Renewable Energy Laboratory, Golden, CO, United States; ^2^Department of Forest Ecology and Genetics, INIA-CIFOR, Madrid, Spain; ^3^Department of Biology, West Virginia University, Morgantown, WV, United States; ^4^Biosciences Division, Center for Bioenergy Innovation, Oak Ridge National Laboratory, Oak Ridge, TN, United States

**Keywords:** biomass cell wall composition, high-throughput analysis, pyrolysis-molecular beam mass spectrometry, bioenergy, glucose, xylose, heritability

## Abstract

The rapid analysis of biopolymers including lignin and sugars in lignocellulosic biomass cell walls is essential for the analysis of the large sample populations needed for identifying heritable genetic variation in biomass feedstocks for biofuels and bioproducts. In this study, we reported the analysis of cell wall lignin content, syringyl/guaiacyl (S/G) ratio, as well as glucose and xylose content by high-throughput pyrolysis-molecular beam mass spectrometry (py-MBMS) for >3,600 samples derived from hundreds of accessions of *Populus trichocarpa* from natural populations, as well as pedigrees constructed from 14 parents (7 × 7). Partial Least Squares (PLS) regression models were built from the samples of known sugar composition previously determined by hydrolysis followed by nuclear magnetic resonance (NMR) analysis. Key spectral features positively correlated with glucose content consisted of *m/z* 126, 98, and 69, among others, deriving from pyrolyzates such as hydroxymethylfurfural, maltol, and other sugar-derived species. Xylose content positively correlated primarily with many lignin-derived ions and to a lesser degree with *m/z* 114, deriving from a lactone produced from xylose pyrolysis. Models were capable of predicting glucose and xylose contents with an average error of less than 4%, and accuracy was significantly improved over previously used methods. The differences in the models constructed from the two sample sets varied in training sample number, but the genetic and compositional uniformity of the pedigree set could be a potential driver in the slightly better performance of that model in comparison with the natural variants. Broad-sense heritability of glucose and xylose composition using these data was 0.32 and 0.34, respectively. In summary, we have demonstrated the use of a single high-throughput method to predict sugar and lignin composition in thousands of poplar samples to estimate the heritability and phenotypic plasticity of traits necessary to develop optimized feedstocks for bioenergy applications.

## Introduction

The composition of lignocellulosic biomass cell walls is a crucial factor in the feasibility of a feedstock for use as a renewable source of fuels and chemicals. Lignocellulosic biomass cell walls are composed of biopolymers including cellulose, hemicelluloses, and lignin that could be used to produce bio-derived products. Carbohydrates, including cellulose, hemicelluloses, and pectins, comprise a large fraction of *Populus* wood cell walls (approximately 45% cellulose, 20% hemicelluloses, and 3% pectins) while lignin constitutes the remaining ∼25% (Mellerowicz et al., 2001; Sannigrahi et al., 2010). Cell wall composition is not only a crucial feedstock characteristic due to the number of products that can be obtained through the processing of the lignocellulosic biomass but also because the interaction of these components may affect biomass recalcitrance (Foston et al., 2011; Gilna et al., 2017). Thus, the optimization of biomass composition could be used to improve biomass processing and conversion. To do so, several approaches could be taken in order to control the composition such as plantation management (e.g., logging intervals, watering, or spacing) and genetic modification [through genetic engineering or breeding (Harman-Ware et al., 2021)].

Breeding uses the natural variation within species complexes to attain desirable values of a trait of interest, both mean and variance values of the traits. Aside from the inherent complexity and cost of managing a breeding program, one underlying biological factor is key for success: the traits of interest must be under at least moderate genetic control and not strongly negatively correlated with each other. Previous studies have shown that the heritability of components of wood is moderate to high. A study on *Populus nigra* showed that broad-sense heritability (*H*^2^) values were 0.48, 0.46, 0.58, and 0.70 for C5, C6 sugars, lignin, and syringyl/guaiacyl (S/G) ratio units in lignin, respectively (Guerra et al., 2013). More recently, our study that controlled for technical and micro-spatial error on several controlled crosses in *Populus trichocarpa* were similar: *H*^2^ was 0.56 for lignin content and 0.81 for the S/G ratio (Harman-Ware et al., 2021). Correlations between C5 and C6 sugars, lignin content, and S/G ratio have been observed in *P. trichocarpa* (Guerra et al., 2016; Happs et al., 2021); for example, lignin and the S/G ratio displayed a moderate positive correlation (*r*_*g*_ = 0.37). Other phenotypes such as enzymatic sugar release (a biomass recalcitrance metric) have also shown correlations with biomass composition phenotypes such as S/G ratio, as demonstrated recently in willow (*r*_*p*_ = ∼0.4) (Ohlsson et al., 2019).

Another approach to feedstock improvement is to identify the loci that control variation in lignocellulosic biomass composition and then specifically target those through breeding or genetic engineering. Genome-wide association studies (GWAS) and quantitative trait loci (QTL) mapping have been used to identify genes associated with wood anatomical and morphological traits (including growth and composition) in various types of *Populus* (Porth et al., 2013; Muchero et al., 2015; Fahrenkrog et al., 2017; Chhetri et al., 2020). Similar to heritability and breeding studies, GWAS and QTL analyses require large populations and replication to maximize diversity, statistical power, resolution, and accuracy of resulting maps and associations. Therefore, an important technical factor needs to be considered: reliable and affordable phenotyping procedures are required to guide breeding and genetic association processes.

Currently, there is a need to utilize rapid techniques capable of analyzing large datasets to determine the sugar composition derived from cellulose and hemicelluloses in biomass in an effort to inform systems biology models, to develop sustainable and consistent feedstocks, and to inform field-to-fuel insights to track changes in biomass composition. The high-throughput analysis of cell wall sugars in lignocellulosic biomass is difficult to achieve as typical methodologies require many steps, including hydrolysis, prior to the analysis of released sugars by high-performance liquid chromatography (HPLC) or nuclear magnetic resonance (NMR; Sluiter et al., 2011; Happs et al., 2020). Various types of high-throughput methods have been developed to estimate sugar composition in biomass and typically involve the use of hydrolysis steps, robotics, and plate reading technology (Decker et al., 2018). Gjersing et al. (2013) and Happs et al. (2021) have developed high-throughput methods for the determination of sugar content in biomass by means of hydrolysis followed by the analysis of hydrolyzates using NMR. The NMR analysis of biomass hydrolyzates is capable of estimating the composition of major and minor sugars present in lignocellulosic biomass cell walls but is still limited in throughput by laborious hydrolysis steps prior to the rapid analysis of the products on the spectrometer.

Pyrolysis-molecular beam mass spectrometry (py-MBMS) has also previously been used to estimate the sugar composition of different types of biomass using Partial Least Squares (PLS) models (Sykes et al., 2015). However, mixed species models cannot accurately predict the sugar compositions of large populations of single species sets. Since there is no need to hydrolyze the samples prior to the py-MBMS analysis and less biomass sample is required, this method is advantageous for the high-throughput estimation of biomass sugar composition if improvements in accuracy and precision can be made. Additionally, lignin content and monolignol composition can be simultaneously measured making py-MBMS potentially capable of comprehensive secondary cell wall analysis in lignocellulosic biomass.

In this study, we reported the development of an accurate high-throughput py-MBMS method that was used to determine the glucose and xylose composition of a large set of *P. trichocarpa* natural variants and a large pedigree set of *P. trichocarpa* by means of PLS models constructed from *P. trichocarpa* of varying sugar content and composition. We compared the cell wall composition of the natural variants and the pedigrees as well as the models that are used to predict the sugar compositions, and also reported the heritability of glucose and xylose composition in the pedigree set. We also used py-MBMS to rapidly predict lignin content in the samples, thus reporting the use of a single method to predict cell wall composition of major components in poplar at a rate of approximately 1 min per sample.

## Materials and Methods

### *Populus trichocarpa* Sample Collection

In total, 924 *P. trichocarpa* natural accessions were grown in OR, United States, and sampled as described previously (Muchero et al., 2015; Chhetri et al., 2019; Happs et al., 2020). In brief, increment cores from 3-year-old trees were debarked, dried, and milled. *P. trichocarpa* pedigrees were grown in OR, United States, and collected as previously described for the construction of a separate model and subsequent prediction of the remaining samples of sugar composition.

### Sugar Composition Analysis

Biomass that had been dried, debarked, milled, and sieved to −20/+80 mesh, ethanol extracted, and destarched was used to determine cell wall sugar composition using high-throughput hydrolysis followed by the NMR analysis of hydrolyzates based on methods described previously (Sluiter et al., 2011; Gjersing et al., 2013; Happs et al., 2021). This method was chosen as it was able to quickly obtain the sugar composition of biomass to build models for sugar prediction by py-MBMS and for the validation of sugar composition estimates. In brief, biomass was hydrolyzed using two-stage acid hydrolysis with H_2_SO_4_, neutralized with CaCO_3_, and filtered. The liquid hydrolyzate was added to D_2_O with a final concentration of 0.01 mg/ml TSP-d_4_ for ^1^H NMR analysis. The ^1^H NMR analysis was conducted on a Bruker Avance III spectrometer at 14.1 T (600.16 MHz) using the following experimental parameters: NOESY-1D with presaturation for water suppression, 5-s recycle delay, and 64 scans. The spectrometer was equipped with a SampleJet sample changer and a Bruker 5-mm BBO probe. Sugar composition by the NMR analysis of hydrolyzates was achieved using PLS modeling approaches described previously (Happs et al., 2021). Notably, 93 samples representing a range of sugar composition were selected from the 924 natural accessions to use as calibration samples to create a model using py-MBMS spectral data. The remaining accessions were analyzed by hydrolysis followed by NMR using the same methodology to validate the sugar composition determined by the py-MBMS analysis. Additionally, the 14 parents plus 10–20 progeny from each of the seven maternal half-sib families were selected, for a total of 121 samples from the 7 × 7 cross pedigree (Supplementary Table 1). These were used for the analysis of sugars by hydrolysis and NMR based on the py-MBMS analyses to cover a range of lignin and sugar-derived ion abundances.

### Pyrolysis-Molecular Beam Mass Spectrometry Analysis

A Frontier PY2020 unit pyrolyzed 4 mg of biomass that had been dried, debarked, milled, and sieved to +80/−20 mesh, ethanol extracted, and destarched. Pyrolysis occurred at 500°C for 30 s (analysis took about 1 min total to analyze a single sample) in 80-μl deactivated stainless steel cups, and each sample was analyzed in duplicate. An Extrel Super-Sonic MBMS Model Max 1000 was used to collect mass spectra, which was processed using Merlin Automation software (V3). Spectra were collected from *m/z* 30 to 450 at 17 eV and mean normalized or total ion chromatogram (TIC) normalized for data analysis and composition prediction. Lignin content was estimated as described elsewhere using a standard of known Klason lignin content and comparing samples based on the summation of ion intensities of *m/z* 120, 124 (G), 137 (G), 138 (G), 150 (G), 152, 154 (S), 164 (G), 167 (S), 168 (S), 178 (G), 180, 181, 182 (S), 194 (S), 208 (S), and 210 (S) where monolignol S/G ratio was calculated by dividing the sum of (S) ions by the sum of (G) ions (Sykes et al., 2008, 2009; Decker et al., 2018). Xylan content was estimated by the use of a PLS regression model that was built using 93 samples whose xylose content was previously determined by the high-throughput NMR analysis of two-stage acid hydrolysis. Additionally, xylose was estimated by the summation of ion intensities of C5 ions *m/z* 57, 73, 85, 96, and 114. Glucose content was estimated by the summation of C6 ions *m/z* 57, 60, 73, 98, 126, and 144 and also determined by PLS regression models built using the data from the high-throughput NMR analysis of hydrolyzates.

### Partial Least Squares Regression Models and Other Data Analyses

The PLS models were constructed using sugar composition data obtained from the NMR analysis of biomass hydrolysis from 93 natural variants of *P. trichocarpa* samples to predict sugar composition in the natural variant population. Natural variant models were cross-validated using both the 93 calibration/model samples and were also later validated on the remaining >800 samples by hydrolysis followed by the NMR analysis of hydrolyzates. PLS models were also separately constructed using hydrolysis followed by the NMR analysis for sugar composition from a pedigree set consisting of 121 samples to predict sugar composition in the remaining ∼2,600 pedigree samples. The 121 pedigree samples were used to validate the sugar models, but further validation by the hydrolysis-NMR analysis of the remaining samples was not possible due to the substantial size of the population. The Unscrambler X version 10.5 was used to build PLS models for py-MBMS spectra from *m/z* 30 to 450. Glucose models were constructed from 4-factor models, and xylose models were constructed from 5-factor models. Other methods of data analysis including descriptive statistics, principal component analysis, and so on were performed using The Unscrambler X version 10.5 and using R Studio (R Core Team, 2013).

## Results

### Glucose and Xylose Models and Contents in *P. trichocarpa* Natural Variants

The *P. trichocarpa* natural variants analyzed by NMR for sugar composition are described in detail by Happs et al. (2021). In brief, the glucose content of the set ranged from approximately 43 to 57% of dry weight (DW) biomass [average of 48 DW%, glucose NMR model root mean square error (RMSE) = 0.01 mg glucose/mg biomass], and the xylose content ranged from 11 to 20% DW biomass (average 17 DW%, xylose NMR model RMSE = 0.01 mg xylose/mg biomass). Of note, 93 training samples for the construction of the py-MBMS PLS model ranged in the glucose content of 43–54% (average 48%) and in the xylose content of 12–20% (average 17%). Previously, C5 (primarily xylose) and C6 (primarily glucose) contents in biomass have been estimated using the py-MBMS data by comparing the relative abundance of C5 and C6 ions described previously, to reference materials of known sugar composition (Sykes et al., 2015). However, this method (reduced-ion, single-point, or response factor comparison) is not sufficient for the estimation of glucose and xylose content in a large sample set of a single biomass type as validated using hydrolysis followed by the NMR analysis (Supplementary Figure 1). The *R*^2^ for the reduced-ion single-point comparison method for xylose content was 0.05 and for glucose content was 0.22 using data from the entire (>900) sample set. Since this simplified ion method is not accurate for the analysis of a single biomass type, PLS models were constructed using glucose and xylose contents determined by hydrolysis followed by the NMR analysis to predict the content of these components in *P. trichocarpa* based on the py-MBMS data.

**FIGURE 1:**
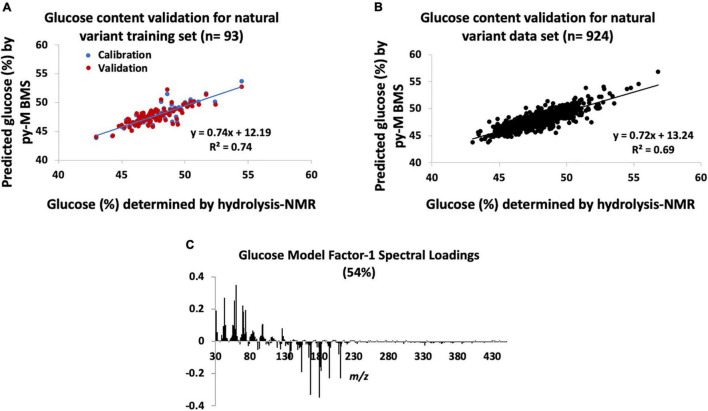
Natural variant glucose models constructed from hydrolysis-nuclear magnetic resonance (NMR) and pyrolysis-molecular beam mass spectrometry (py-MBMS) spectra and validation. **(A)** Glucose content validation for natural variant training set, **(B)** glucose content validation for all natural variants tested, and **(C)** factor-1 spectral loadings for natural variant glucose model.

The errors associated with the PLS model used to determine the glucose content of the natural variant poplar samples using py-MBMS spectra are outlined in Supplementary Table 2 (RMSE of the py-MBMS glucose model was 0.01 mg glucose/mg biomass, total average error including NMR and MBMS error = 0.03 mg glucose/mg biomass). The training set had *R*^2^ = 0.74 for the calibration of measured and predicted values and had Pearson’s correlation coefficient (PCC) of 0.86 (Figure 1A). The error in the values of the training samples ranged from −5.8 to +5.7% (relative to the value) with an average error of | 1.4%| (SD = 1.2%) (Supplementary Table 2). The validation of the glucose content estimates for the full natural variant sample set (*n* = 924) by the NMR analysis of hydrolyzates based on the model constructed of the 93 samples resulted in larger errors (Supplementary Table 2 and Figure 1B).

Table 1 lists the ions with the highest correlation to glucose content (also refer to model correlation Factor 1 loadings, Figure 1C). Several of the ions with the highest correlation coefficients to glucose content have previously been associated with estimating glucose and C6 content of biomass (Sykes et al., 2015). However, additional ions that have also been attributed to sugar-derived pyrolyzates (Evans and Milne, 1987; Sykes et al., 2015) were also among those most strongly correlated with glucose abundance. These glucose-derived ions were also generally negatively correlated with ions derived from lignin (Figure 1C) including *m/z* 154 (S), 167 (S), 180, 194 (S), and 210 (S) (e.g., PCC for glucose content determined by NMR and S-derived lignin ions is approximately −0.5). There was no strong correlation observed between the S/G ratio and the glucose content (PCC = −0.2). The contribution of ions 69, 70, 84, and potentially 96 is likely important for predicting glucose content, particularly in comparison with the single-point comparison method previously reported. These findings indicate that the simplified ion summation with single-point response prediction previously used for estimating C6 content did not consist of all ions of interest needed for accurate analyses.

**TABLE 1:** Pearson’s correlation coefficients (PCCs) for selected ions as they relate to glucose content in *P. trichocarpa* natural variant biomass samples.

*m/z*	Pearson’s correlation coefficient – glucose content	Known source
126*	0.69	C6
98*	0.64	C6
69	0.58	C5, C6
70	0.57	C5, C6
84	0.57	C6
57*	0.44	C5, C6
60*	0.38	C5, C6
73*	0.36	C5, C6
144*	0.28	C6
96	0.25	C5, C6

*Reduced-ion single-point comparison method to estimate glucose content sums and compares the intensities of m/z 57, 60, 73, 98, 126, and 144.

**FIGURE 2:**
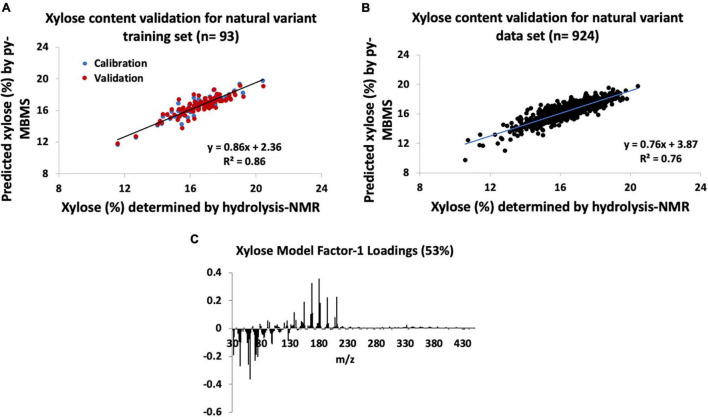
Natural variant xylose models constructed from hydrolysis-NMR and py-MBMS spectra and validation. **(A)** Xylose content validation for natural variant training set, **(B)** xylose content validation for all natural variants tested, and **(C)** factor-1 spectral loadings for natural variant xylose model.

The py-MBMS PLS model used to determine the xylose content of the natural variant training poplar samples (*n* = 93) had *R*^2^ = 0.86 for the calibration of measured and predicted values, and errors are outlined in Supplementary Table 3 and shown in Figure 2A. RMSE of the py-MBMS xylose model was 0.004 mg xylose/mg biomass, when combined with the error of NMR model = 0.05 mg xylose/mg biomass total error. The predicted and measured xylose content of the training set had a PCC of 0.93. While the xylose content estimates of the natural variant set had higher *R*^2^ and correlation coefficients for the training set and entire validation set (Figure 2B) in comparison with the glucose content estimates, the range of error of xylose estimates was substantially higher. Similar to glucose estimates, the errors associated with the entire set based on the model constructed from the training set were substantially higher.

Interestingly, ions with the highest correlation to xylose content were primarily attributed to lignin-derived species, including *m/z* 165, 180, 168, and 167 (Table 2 and Figure 2C). Ions previously used to estimate xylose content and otherwise known to derive from sugars actually had a negative correlation with xylose content, with an exception for *m/z* 114 which only moderately correlated with xylose content. The positive correlation between xylose and lignin content likely has genetic origins related to carbon allocation and may not necessarily be extrapolated to other biomass types and may also be a reason for the higher errors observed for xylose content determination. There was no strong correlation observed between S/G and xylose content (PCC = 0.2). Additionally, these findings also support the need for PLS models to more accurately predict C5 sugars such as xylose content in a single biomass type in comparison with the previously used simplified ion summation method.

**TABLE 2:** PCCs for selected ions as they relate to xylose content in *P. trichocarpa* natural variant biomass samples.

*m/z*	Pearson’s correlation coefficient – xylose content	Known source
Σ lignin ions	0.54	lignin
165	0.54	S lignin
180	0.52	lignin
168	0.50	S lignin, vanillic acid
167	0.47	S lignin
153	0.47	S lignin, vanillic acid
114*	0.32	C5
150	0.28	C5, lignin, ferulate
103	0.27	C5
57*	−0.41	C5, C6
73*	−0.34	C5, C6
85*	−0.04	C5, C6
96*	−0.23	C5, C6

*Reduced-ion single-point comparison method for the estimation of xylose content sums and compares the intensities of m/z 57, 73, 85, 96, and 114.

### Glucose and Xylose Models and Contents in *P. trichocarpa* Pedigrees

**FIGURE 3:**
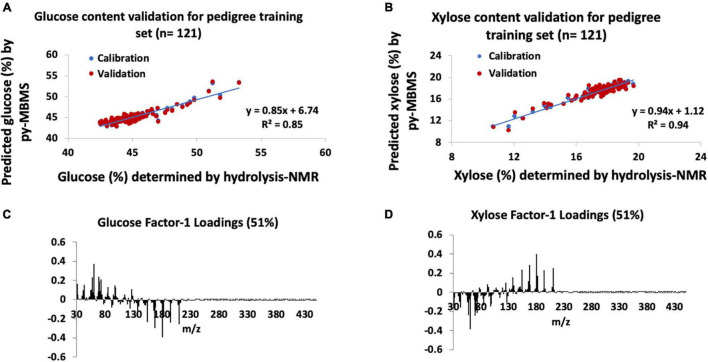
Models for glucose and xylose content estimates in pedigrees of *P. trichocarpa*. **(A)** Glucose content validation for pedigree training set, **(B)** xylose content validation for pedigree training set, **(C)** factor-1 spectral loadings for pedigree glucose model, and **(D)** factor-1 spectral loadings for pedigree xylose model.

The py-MBMS PLS model that was used to determine the glucose content of the pedigree poplar samples had *R*^2^ = 0.85 (Figure 3A) for the calibration of measured and predicted values, and the error ranged from −3.8 to +5.8% of the value with an average error of | 1.3%| (SD = 1.0%, *n* = 121). The predicted and measured glucose content of the training set had a PCC of 0.92. RMSE of both the NMR and py-MBMS models for glucose prediction was 0.01 mg glucose/mg biomass, for a total error of 0.03 mg glucose/mg biomass. The validation of the glucose (and xylose) content estimates was not established for all samples due to the size of this sample set (an additional 2,600 test samples in addition to the training samples), although the high degree of correlation and relatively low error range of the training set indicated reasonable accuracy of glucose prediction of this sample set.

Similar to the natural variant set, the model constructed for xylose content estimates of the pedigree set had higher *R*^2^ and PCCs as well as higher error ranges in comparison with the glucose models. The validation of the training set (*n* = 121) of pedigree samples for xylose content (Figure 3B) had *R*^2^ = 0.94 for the calibration of measured and predicted values with a PCC of 0.97. The xylose content error of the training set ranged from −6.3 to +6.3% of the value with an average error of | 1.8%| (SD = 1.5%). RMSE of the py-MBMS xylose model was 0.004 mg xylose/mg biomass, when combined with the error of NMR model = 0.05 mg xylose/mg biomass total error. In summary, the PLS models for xylose prediction were acceptable for the training set in the pedigrees although the remaining samples in the set could not be validated.

**FIGURE 4:**
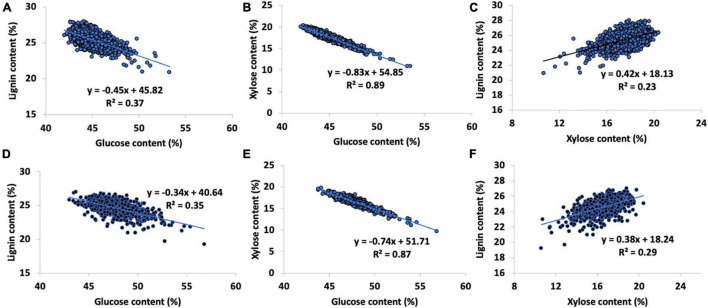
Relationships between biopolymer components in the pedigree [(Top, **A**) lignin vs. glucose content, **(B)** glucose vs. xylose content, **(C)** lignin vs. xylose content] and natural variant [(Bottom, **D**) lignin vs. glucose content, **(E)** glucose vs. xylose content, and **(F)** lignin vs. xylose content] *P. trichocarpa* sets. Natural variant sugar contents shown used hydrolysis-NMR validation data to minimize propagated error in the py-MBMS data.

Also similar to the natural variants, Factor 1 loadings for the model for glucose (Figure 3C) consisted of ions including *m/z* 60, 69, 73, 98, and 126 were positively correlated with glucose content whereas lignin-derived ions such as *m/z* 154 (S), 167 (S), 180, 194 (S), and 210 (S) are negatively correlated with predicted glucose content (PCC with S-derived ion = −0.4) and glucose-derived ions, although there was no correlation observed between S/G and glucose content (PCC = 0). Xylose content in the pedigree samples, such as the natural variants, also confirmed a positive correlation between xylose content and lignin-derived species [including *m/z* 124 (G), 137 (G), 154 (S), 167 (S), 180, 194 (S), and 210 (S), refer to Figures 3D, 4C,F and Supplementary Figure 2], although there was no correlation observed between S/G and xylose content (PCC = 0).

The glucose and xylose content predictions of the pedigree set were strongly negatively correlated (*R*^2^ = 0.89, PCC = 0.94, Figure 4B). Glucose and lignin contents were moderately negatively correlated (PCC = −0.61, *R*^2^ = 0.37, Figure 4A) while xylose and lignin contents were weakly positively correlated (PCC = 0.48, *R*^2^ = 0.23, Figure 4C). An average of 88 wt% of the mass of the material in the pedigree set was accounted for in glucose, xylose, and lignin contents. The remaining mass was likely metabolites, particularly phenolics and salicylates (Harman-Ware et al., 2021), free sugars and other carbohydrates not accounted for (including pectins), as well as inorganic ash components and proteins.

### Comprehensive Composition of *P. trichocarpa* Natural Variants

The py-MBMS analysis of the *P. trichocarpa* natural variants was also used to determine the lignin content and lignin monomeric S/G ratios of the samples, where the lignin information of a subset of these samples was provided in the previous study (Happs et al., 2021). The lignin content and S/G variation of the natural variants are shown in Table 3 and are similar to that of the pedigree set [i.e., the extensive analysis of the lignin content, S/G ratio, and corresponding ions in the pedigree set is reported in Harman-Ware et al. (2021)]. The principal component analysis of the natural variant spectra shows that the majority of the variance is explained by a negative relationship between lignin and sugar-derived ions [i.e., the first principal component (PC-1) explains 57% variance, refer to Supplementary Figures 2, 3]. The second principal component (PC-2), explaining 15% of the variance, shows variance generally attributed to a positive correlation in C5 sugar-derived ions and S-lignin-derived ions, together negatively correlated with G-lignin ions.

**TABLE 3:** Lignin content and lignin syringyl/guaiacyl (S/G) ratio determined by the py-MBMS analysis of *P. trichocarpa* natural variants.

	S/G	Lignin content (%)
Mean	2.1	24.6
Max	2.6	27.0
Min	1.4	19.3
Range	1.1	7.8
Std. deviation	0.1	1.0

Since the full natural variant set was validated using the NMR data, other sugars including mannose, arabinose, and galactose were fully accounted for, although these sugars occurred in lower abundance and were not able to be predicted using the py-MBMS data. The total sum of material averaged 95% recovery, indicating that a large amount (approximately 7 wt%) of the missing pedigree mass could be explained by the abundance of minor sugars. There was a strong negative correlation in the glucose and xylose composition of the natural variant *P. trichocarpa* set (using hydrolysis NMR data to minimize error propagation, PCC = −0.76, *R*^2^ = 0.87, Figure 4E; Happs et al., 2021) and a slight negative correlation between lignin content and glucose content, *R*^2^ = 0.35, (Figure 4D). Lignin ions, particularly S-derived such as *m/z* 154, 194, and 210 and the predicted xylose content from the PLS models using MBMS data, showed a weak positive correlation (using the sum of S-derived ions, PCC = 0.47, *R*^2^ = 0.22, Supplementary Figure 3 and Figure 4F).

### Heritability of Sugars in *P. trichocarpa*

The comprehensive py-MBMS spectral analysis of the pedigree set with a particular focus on lignin content and composition was described previously (Harman-Ware et al., 2021). Table 4 highlights the broad-sense heritability of ions positively correlated with glucose and xylose contents (or more broadly, known to originate from C6 to C5 sugars) and the broad-sense heritability of those sugars based on estimates by the py-MBMS analysis after spectral correction for the microspatial variation of the individuals and for the instrumental variation. Prior to the removal of the microspatial and instrumental variation, the broad-sense heritability of the predicted sugars was lower for glucose (0.31 before and 0.32 after spectral adjustments accounting for the microspatial and instrumental variance) and slightly higher for xylose (0.36 before and 0.34 after). The reduced xylose broad-sense heritability after spectral data correction may originate from many sources which will be discussed later; and the increased heritability for glucose is consistent with the increase in lignin heritability (Harman-Ware et al., 2021). However, the differences may be considered minor (within error), and the broad-sense heritability discussed here focuses on the values obtained after the microspatial and instrumental variance correction. Of the sugar-derived ions, *m/z* 60 had the highest heritability, and all ions in Table 4 required thin-plate spline (TPS) correction, indicating that these ions and their corresponding biomass components (sugars) were impacted by microspatial variation in the field, and hence, the sugar contents exhibited some phenotypic plasticity (Harman-Ware et al., 2021). The heritability of glucose and xylose contents was 0.32 and 0.34, respectively, and the heritability of sugar-derived ions was generally lower than that of lignin and phenolic-derived ions in *P. trichocarpa*, which ranged from 0.31 to 0.79 (Harman-Ware et al., 2021).

**TABLE 4:** Broad-sense heritability of glucose and xylose contents and ions positively correlated with glucose and xylose contents and annotated based on the py-MBMS analysis of the *P. trichocarpa* pedigree set [heritability of ions and annotations summarized from Harman-Ware et al. (2021)].

*m/z*	Source	Heritability
57	C5 and C6	0.28
60	C5 and C6	0.35
69	C5 and C6	0.29
70	C5 and C6	0.32
73	C5 and C6	0.35
84	C6	0.13
85	C5 and C6	0.21
96	C5 and C6	0.13
98	C6	0.23
103	C5	0.13
114	C5	0.34
126	C6	0.22
144	C6	0.05
Glucose content		0.32
Xylose content		0.34

## Discussion

### Models and Sugar Analysis Methodology

The py-MBMS analysis of hardwood biomass for sugars is rapid but requires the use of a large number of calibration samples with *a priori* sugar compositional analysis and construction of PLS models. While previous studies using different biomass types demonstrated the potential use of py-MBMS for C5 and C6 estimates using simplified ion summation methods, those techniques were not accurate for the analysis of a large sample set of a single biomass type (Supplementary Figure 1). Differences between the models from the natural variant population and the pedigree samples were minimal although the accuracy was overall greater for the pedigree samples. The higher accuracy of the pedigree models likely results from higher uniformity in the population composition and the use of a larger calibration set. Errors in the predicted major sugar composition could result from the relative abundance of lignin, celluloses, and hemicelluloses where the less abundant sugars in the cell walls as well as inorganic components impact the product distribution of sugar pyrolysis and may contribute to spectral features not fitting within the model ranges. Limitations to this methodology also include the indirect measurement methodology, requiring maintenance of the calibration, as well as the ability to obtain enough representative feedstocks of known sugar composition for model training. However, these results outline a reasonable method for the high-throughput analysis of all major secondary cell wall biopolymers in lignocellulosic biomass by py-MBMS.

### Relationships Between Biopolymers in Biomass

Both the natural variants and the pedigree samples exhibited a strong negative relationship between glucose and xylose content (Figures 4B,E). While this observation may be partially due to artifacts in the models (potentially in the NMR data as well), it is consistent across the sets, and the relationships between xylose and lignin provide further insights. For example, the model predicting xylose content consists of a significant contribution of lignin-derived ions; however, the relationships between lignin and xylose content predictions show weak correlations (*R*^2^ = 0.23 and 0.29 for pedigree and natural variants, respectively). Our observations are in contrast to those observed previously, likely due to the fact that C5 and C6 sugars were previously calculated using the simplified ion method (Guerra et al., 2016). The heritability of xylose and glucose contents estimated here are also similar or higher than previously reported, again due to the improvement in the accuracy of the measurements based on the models used as well as the correction of the data to account for microspatial variation in the field. However, the heritability of the xylose values using uncorrected spectral data was slightly higher, potentially due to the inclusion of confounding ion intensities that are partially sourced from the components of higher heritability. There may also be differential phenotypic plasticity that would explain a higher broad-sense heritability of xylose content prior to the microspatial correction of the data. The relationships between and the heritability of the biomass components including glucose, xylose, and lignin content as well as S/G are very important for the design of bioenergy crops as these components directly impact the economics associated with the conversion of biomass to fuels, materials, and energy (Happs et al., 2021; Harman-Ware et al., 2021). Additionally, the relative abundance of biomass components may also play important ecological roles and impact the sustainability associated with a given crop.

## Conclusion

Multivariate models need to be constructed to predict glucose and xylose contents present in the cellulose and hemicellulose biopolymers in cell walls for the py-MBMS analysis of large sets of *P. trichocarpa*. Models constructed from different training sets confirm the relationships between specific ions and sugar sources as well as the relationships between different biopolymers in *P. trichocarpa*. The py-MBMS was able to rapidly (approximately 1 sample/min) determine the contents of all the major cell wall components in *P. trichocarpa* including glucose, xylose (from cellulose and hemicelluloses, respectively), and lignin contents as well as lignin S/G ratios in order to inform the variability and heritability of biomass cell wall compositional phenotypes. The heritability of sugar contents in *P. trichocarpa* is lower than that of lignin content and lignin monomeric ratios based on py-MBMS analyses. These results show that we can use a single high-throughput method to measure biomass composition to identify the relationships between biopolymers in natural variants and pedigrees of *P. trichocarpa* which could potentially be leveraged to design *P. trichocarpa* crops of specific compositions to optimize economics, conversion, and sustainability metrics. It will be important to understand the relationships between biopolymer and cell wall composition to efficiently domesticate the lignocellulosic crops for the large-scale production of bio-derived products in moving toward a bio-based economy.

## Data Availability Statement

The raw data supporting the conclusions of this article will be made available by the authors, without undue reservation.

## Author Contributions

AH-W performed the py-MBMS experiments, data analysis, and wrote the manuscript. RH performed the NMR analyses and contributed to manuscript text. DM-S prepared samples, performed the data analysis for heritability, and contributed to manuscript text. CD prepared samples and performed the hydrolysis and py-MBMS experiments. WM provided oversight for the natural variant population and edited the manuscript. SD designed the study, provided oversight, and edited the manuscript. All authors contributed to the article and approved the submitted version.

## Author Disclaimer

The views expressed in this study do not necessarily represent the views of the United States Department of Energy or the United States Government.

## Conflict of Interest

The authors declare that the research was conducted in the absence of any commercial or financial relationships that could be construed as a potential conflict of interest.

## Publisher’s Note

All claims expressed in this article are solely those of the authors and do not necessarily represent those of their affiliated organizations, or those of the publisher, the editors and the reviewers. Any product that may be evaluated in this article, or claim that may be made by its manufacturer, is not guaranteed or endorsed by the publisher.
