# Supplementary material for: Abundance of Major Cell Wall Components in Natural Variants and Pedigrees of Populus trichocarpa
Source: Front Plant Sci. 2022 Feb 3;13:757810. doi: 10.3389/fpls.2022.757810 (PMC8850957; doi:10.3389/fpls.2022.757810)
Supplement: Supplementary file 1 [file Table_1.DOCX]

Supplementary Material

# Supplementary Tables

**Supplementary Table 1.** Maternal half-sib family sizes.

| Mother | Fathers | Offspring |
| --- | --- | --- |
| 1863 | 6 | 15 |
| 1909 | 7 | 19 |
| 1950 | 6 | 20 |
| 2048 | 6 | 12 |
| 2066 | 6 | 15 |
| 2283 | 5 | 10 |
| 4593 | 6 | 16 |

**Supplementary Table 2. Glucose content errors based on py-MBMS analysis of natural variant poplar sets.**

|  | Validation training set (n=93) | Validation whole set (n=924) |  |
| --- | --- | --- | --- |
| Error range (%) | -5.8 to 5.7 | -7.5 to 8.5 |  |
| Average error (std dev) (%) | 1.4 (+/- 1.2) | 3.2 (+/- 2.7) |  |
| Pearson correlation coefficient | 0.86 | 0.83 |  |

**Supplementary Table 3. Natural variant xylose content validation errors**

|  | Validation training set (n=93) | Validation whole set (n=924) |  |
| --- | --- | --- | --- |
| Error range (%) | -8.2 to 10.3 | -16.9 to 14.5 |  |
| Average error (std dev) (%) | 2.3 (+/- 2.0) | 3.2 (+/- 2.7) |  |
| Pearson correlation coefficient | 0.93 | 0.87 |  |

# Supplementary Figures

**Supplementary Figure 1.** Reduced ion method used to estimate xylose and glucose content from py-MBMS data.

**Supplementary Figure 2.** Principal component analysis of natural variant *P. trichocarpa* set.

**Supplementary Figure 3.** Natural variant relationship between xylose and S lignin, left; pedigree xylose and S lignin relationships, right.
